# Paraneoplastic pemphigus associated with follicular lymphoma and myasthenia gravis: Efficacy of a lenalidomide-rituximab-corticosteroid regimen

**DOI:** 10.1016/j.jdcr.2026.07.024

**Published:** 2026-07-21

**Authors:** Yiting Shen, Qijun Wang, Chuqiao Xu, Wenqing Zhu, Haiqin Zhu, Meng Pan, Jingying Wang

**Affiliations:** Department of Dermatology, Ruijin Hospital, Shanghai Jiao Tong University School of Medicine, Shanghai, China

**Keywords:** follicular lymphoma, lenalidomide, myasthenia gravis, paraneoplastic pemphigus, rituximab

Paraneoplastic pemphigus (PNP), first characterized in 1990, is a rare and life-threatening, mucocutaneous, autoimmune blistering disease associated with underlying neoplasms.^1^ PNP typically manifests as intractable stomatitis and polymorphous cutaneous eruptions, driven by both humoral and cell-mediated autoimmune responses. Beyond the skin, multiorgan involvement, including bronchiolitis obliterans, ocular complications, and myasthenia gravis (MG), further underscores the systemic nature of the disease. As understanding evolved to recognize PNP as a systemic multiorganopathy, the broader term paraneoplastic autoimmune multiorgan syndrome was proposed.^2^ Here, we present a complex case of PNP/paraneoplastic autoimmune multiorgan syndrome accompanied by MG, follicular lymphoma (FL), and bronchiolitis obliterans, which showed a favorable response to a lenalidomide-rituximab-corticosteroid regimen.

## Case report

**Fig 1:**
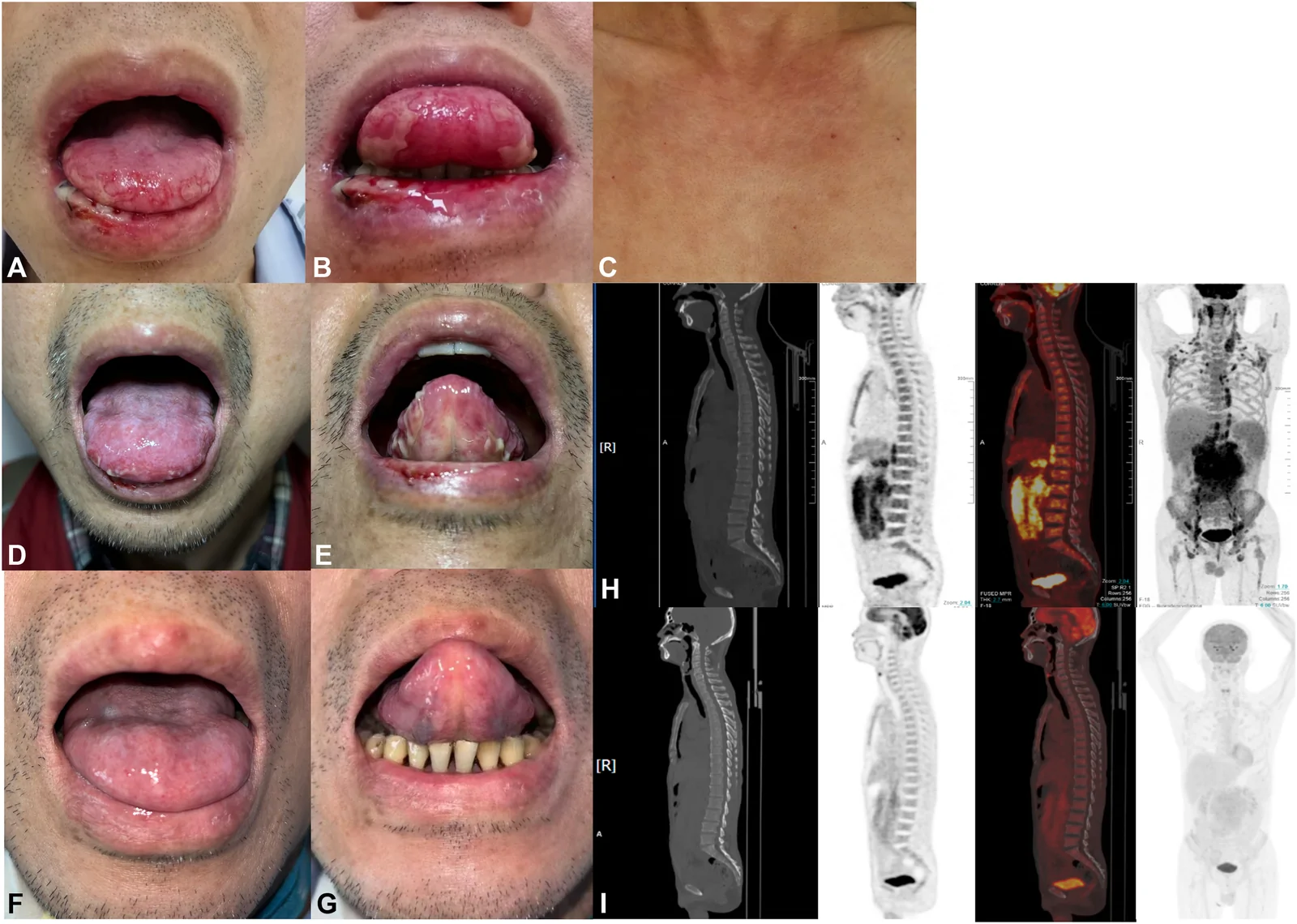
Clinical findings before and after treatment. **A-C,** Pretreatment clinical manifestations showing severe oral and lingual ulcers **(A, B)** and scattered erythematous patches on the trunk **(C)**. **D-G,** Significant resolution of oral and lingual erosions after 3 months **(D, E)** and 6 months **(F, G)** of treatment. **H, I,** Positron emission tomography/computed tomography imaging at baseline **(H)** and follow-up **(I)**, demonstrating partial remission of the underlying follicular lymphoma following R^2^ regimen.

A 49-year-old man was admitted with severe mucosal erosions and bilateral ptosis (Fig 1, *A-C*). Four months prior, he was initially diagnosed with "pemphigus" based on oral ulcers and elevated anti-Dsg3 antibodies. However, the concurrent low-titer positivity for anti-BP180 antibody was overlooked at the time. While his symptoms transiently improved with prednisone, the oral lesions relapsed during tapering, accompanied by a progressive cough and a 2-week history of ptosis. Physical examination revealed extensive erosions of the gingiva, lips, tongue, palate, and glans penis, alongside scattered asymptomatic erythema on the trunk and upper limbs.

**Fig 2:**
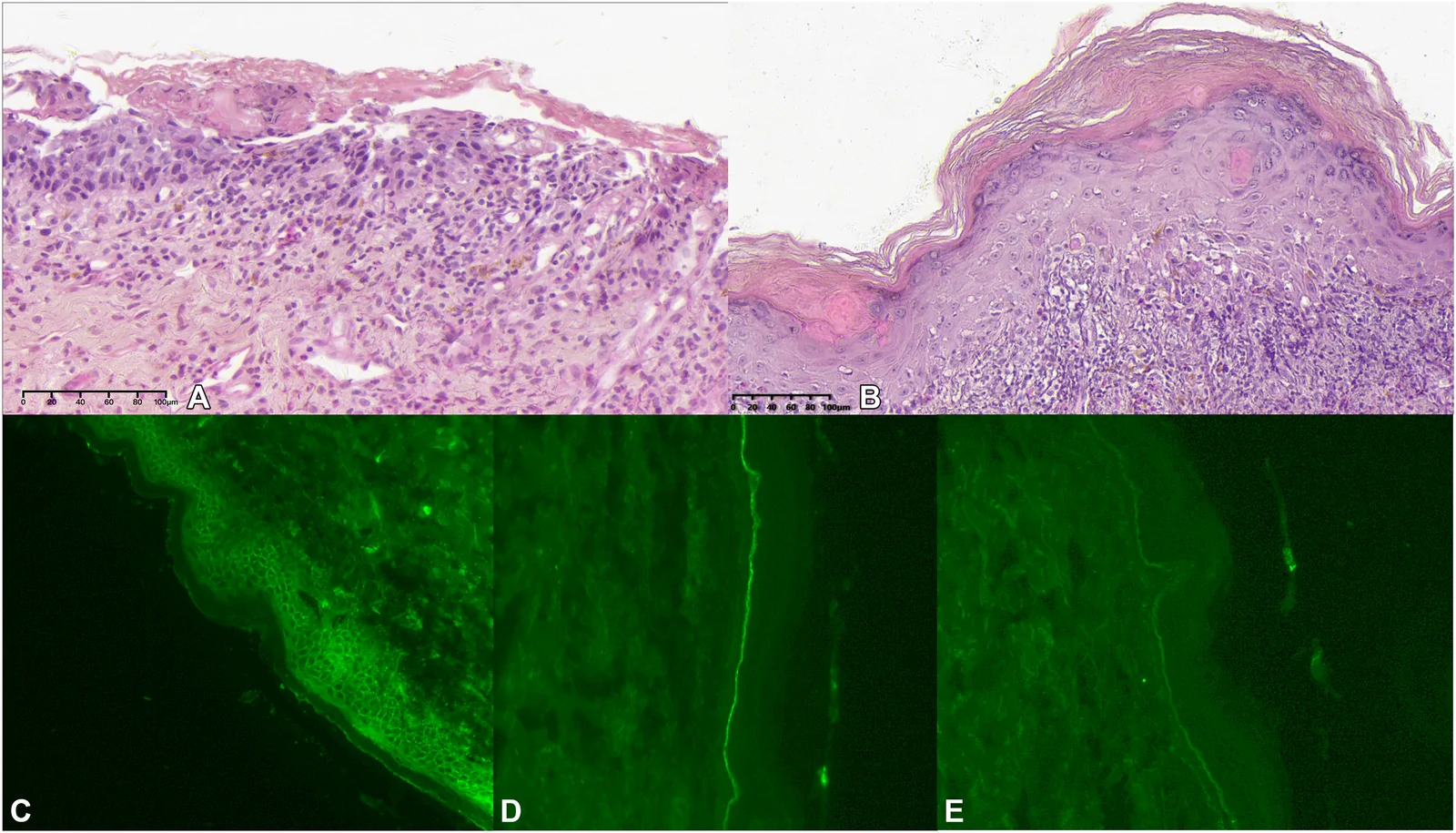
Histopathological findings of the lesions. **A, B,** Hematoxylin and eosin staining of the oral **(A)** and trunk **(B)** lesions showed intraepidermal acantholysis and interface dermatitis with dense infiltration of eosinophils and lymphocytes in the papillary dermis. **C-E,** Direct immunofluorescence revealed weak intercellular IgG deposition **(C)** between the epidermal cells, along with C3^+^**(D)** and weak immunoglobulin M **(E)** deposition at the basement membrane zone.

Blood examination showed persistent anti-Dsg3 positivity (163.72 U/ml; positive >20) and anti-A2ML1 IgG antibody (1:100), while anti-Dsg1 antibody, anti-BP180 antibody, and anti-BP230 antibody test results were within the normal range. Indirect immunofluorescence on rat bladder confirmed the presence of IgG cell-surface antibodies. Punch biopsy of the erythema on the chest showed interface dermatitis with dense infiltration of eosinophils and lymphocytes in the papillary dermis, while the oral mucosal lesions exhibited intraepidermal acantholysis and dyskeratotic cells (Fig 2, *A, B*). Direct immunofluorescence demonstrated extracellular IgG deposition within the epidermis, along with linear C3 and immunoglobulin M deposition at the basement membrane zone (Fig 2, *C-E*). Based on these results, we suspected a diagnosis of PNP.

Further investigation via positron emission tomography/computed tomography revealed systemic lymphadenopathy (Fig 1, *G*). Lymph node and bone marrow biopsies confirmed grade 1 to 2 FL (stage IV). These cells were positive for CD20, CD10, and Bcl-6 but negative for CD3, CD5, CD30, and CyclinD1, with a Ki67 proliferation index of approximately 10%. Regarding the ptosis, MG was diagnosed based on a positive Jolly test, elevated titers of anti-AchR antibody (1:320), anti-Titin antibody (1:100), anti-RyR antibody (1:100), and impaired neuromuscular transmission on electromyogram. The patient, a nonsmoker, presented with a cough and mild dyspnea on exertion. Pulmonary function testing isolated small airway dysfunction (FEF 25-75 and FEF 50 reduced by 50%), which is highly suspicious for bronchiolitis obliterans.

A diagnosis of PNP associated with FL and MG was established. A multidisciplinary team formulated a combined regimen: oral prednisone (40 mg/day), pyridostigmine bromide (30 mg 3 times a day), and inhaled tiotropium bromide. For the underlying lymphoma and paraneoplastic manifestations, the patient received the R^2^ regimen (rituximab 375 mg/m^2^ on day 1 and lenalidomide 25 mg on days 1-10 of a 21-day cycle). Oral and penile erosions, as well as the ptosis, exhibited ongoing improvement in 6 months (Fig 1, *D-G*), and the lymphoma achieved partial remission after 3 treatment cycles (Fig 1, *I*).

## Discussion

PNP is a rare mucocutaneous blistering disease intrinsically linked to underlying malignancies. While pemphigus vulgaris (PV) and PNP may share similar clinical features, PNP is distinguished by its remarkable morphological heterogeneity. Unlike the typical flaccid blisters and erosions seen in PV, PNP presents a diverse spectrum of lesions, including pemphigus-like, pemphigoid-like, erythema multiforme-like, graft-versus-host disease–like, or lichen planus-like lesions.^2^ Initially misdiagnosed as PV, our patient exhibited a hybrid phenotype of pemphigus-like oral erosions and chronic graft-versus-host disease–like cutaneous patches. The presence of PNP-specific antibodies, positive rat bladder indirect immunofluorescence, and immunoglobulin deposition within the epidermal intercellular spaces and linearly along the basement membrane zone on direct immunofluorescence were pivotal in re-evaluating the diagnosis. Early recognition of PNP is critical. We recommend maintaining a high index of suspicion for malignancy in the setting of atypical lesions or treatment-refractory disease, especially those with dual-pattern direct immunofluorescence.

The coexistence of PNP, MG, and FL is rare and suggests a broad-spectrum failure of immune tolerance. In a cohort of 106 patients with PNP, it is noteworthy that overall 39% of patients with PNP experienced muscle weakness, and 35% were diagnosed with MG, with Castleman disease and thymoma being the most frequently associated tumors.^3^ Our case is particularly rare because of its association with FL. It has been established that tumor B-cell clones serve as a reservoir for autoantibody production in PNP with Castleman disease.^4^ Although the exact mechanism needs further investigation, we hypothesize that the aberrant immune response driven by B-cell clones in FL may be associated with the production of diverse autoantibodies targeting the skin, mucosa, and neuromuscular junctions.

Treatment of PNP remains challenging, requiring a dual strategy of eradicating the underlying malignancy and suppressing the life-threatening autoimmune response. Rituximab, an anti-CD20 monoclonal antibody, is the standard of care for both FL and antibody-mediated autoimmune diseases; it depletes the B-cell clones responsible for autoantibody production. In patients with FL, rituximab combined with chemotherapy (eg, R-CHOP) is established as a first-line therapy^5^ and has also been reported to benefit patients with PNP.^6^ However, conventional chemotherapy is frequently associated with significant cytotoxic adverse effects, including myelosuppression, severe infections, and gastrointestinal and cardiac toxicities. To mitigate these risks, we explored a chemotherapy-free approach using lenalidomide. As an immunomodulatory agent, lenalidomide degrades transcription factors Aiolos and Ikaros, inducing apoptosis in malignant B-cells while modulating T cell function.^7^ Although the use of lenalidomide in PNP is unreported, evidence from our previous study supports its potential utility: thalidomide (a lenalidomide analog) exhibited promising efficacy and safety as an adjuvant therapy in a cohort of 14 patients with PNP.^8^ Furthermore, lenalidomide has demonstrated clinical benefits in managing refractory oral erosions in Behçet disease^9^ and PV.^10^ This combination achieved significant improvement in both the oncological status and paraneoplastic symptoms. Compared to traditional chemoimmunotherapy, the R^2^ regimen may offer a superior safety profile, with no adverse events observed in our patient, suggesting it may be a viable and less toxic alternative for PNP management.

## Conflicts of interest

None disclosed.

## References

[1] Anhalt G.J., Kim S.C., Stanley J.R. (1990). Paraneoplastic pemphigus. An autoimmune mucocutaneous disease associated with neoplasia. N Engl J Med.

[2] Anderson H.J., Huang S., Lee J.B. (2024). Paraneoplastic pemphigus/paraneoplastic autoimmune multiorgan syndrome: Part I. Clinical overview and pathophysiology. J Am Acad Dermatol.

[3] Wang R., Li J., Wang M. (2015). Prevalence of myasthenia gravis and associated autoantibodies in paraneoplastic pemphigus and their correlations with symptoms and prognosis. Br J Dermatol.

[4] Wang L., Bu D., Yang Y., Chen X., Zhu X. (2004). Castleman's tumours and production of autoantibody in paraneoplastic pemphigus. Lancet.

[5] Marcus R., Imrie K., Belch A. (2005). CVP chemotherapy plus rituximab compared with CVP as first-line treatment for advanced follicular lymphoma. Blood.

[6] Barnadas M.A., Roe E., Brunet S. (2006). Therapy of paraneoplastic pemphigus with rituximab: a case report and review of literature. J Eur Acad Dermatol Venereol.

[7] Gandhi A.K., Kang J., Havens C.G. (2014). Immunomodulatory agents lenalidomide and pomalidomide co-stimulate T cells by inducing degradation of T cell repressors Ikaros and Aiolos via modulation of the E3 ubiquitin ligase complex CRL4(CRBN.). Br J Haematol.

[8] Wang J., Zhang Y., Pan M. (2020). Thalidomide as a potential adjuvant treatment for paraneoplastic pemphigus: a single-center experience. Dermatol Ther.

[9] Green J., Upjohn E., McCormack C., Zeldis J., Prince H.M. (2007). Successful treatment of Behçet’s disease with lenalidomide. Br J Dermatol.

[10] Jain A.K., Agarwal S., Gautam M. (2022). Successful management of severe recalcitrant pemphigus vulgaris with lenalidomide. Indian Dermatol Online J.

